# A Comparative Study of Functional Outcomes in Unstable Pelvic Ring Fractures Treated With Internal Fixator (INFIX) With and Without Sacroiliac Joint Screw Fixation

**DOI:** 10.7759/cureus.60279

**Published:** 2024-05-14

**Authors:** Muqtadeer Ansari, Vimal P V, Arpit K Kesharwani, Saurabh S Damkondwar, Rohan U Kakade

**Affiliations:** 1 Department of Orthopedics, Government Medical College Aurangabad, Aurangabad, IND

**Keywords:** infix, anterior pelvic ring injury, sacroiliac joint screw, unstable pelvic ring injury, traumatic pelvic fractures

## Abstract

Introduction

Despite constituting only 3-8% of orthopedic trauma cases, pelvic injuries are associated with high mortality rates, emphasizing the critical need for stable fixation rather than merely anatomical alignment. The use of an anterior, subcutaneous, internal pelvic fixator (INFIX), a novel technique, has shown promise in treating these injuries. Posterior pelvic ring injuries are challenging because they require a significant level of surgical training and technical expertise, and each treatment method has disadvantages. The aim of this study is to compare the clinical and biomechanical outcomes of INFIX with and without sacroiliac screw fixation for unstable pelvic fractures.

Methods and methodology

Retrospectively, we selected 20 patients with unstable pelvic ring injuries who had come to a high-volume tertiary care hospital and medical college in the state of Maharashtra, India. All the patients were operated on with INFIX; 10 with a sacroiliac joint screw and 10 without a sacroiliac joint screw. We followed up with the patients for six months and evaluated them according to the Majeed score.

Results

Functional outcomes differed little between INFIX patients operated on with and without a sacroiliac joint screw. However, morbidity, hospital stay, the need for ICU, radiation exposure, and technical ease of surgery were improved in INFIX patients without the sacroiliac screw procedure. We noted an average Majeed score of 78 in the INFIX-alone group and 77.2 in the group that received INFIX with a sacroiliac joint screw. Six months after the surgery, the patients showed signs of a stable bony union, had achieved a full range of motion, and reported no problems in their day-to-day work.

Conclusion

Although this was a short-term study, we conclude that INFIX without a sacroiliac joint screw showed a comparable functional outcome compared to INFIX with a sacroiliac joint screw. Patients with INFIX alone showed better results; they had reduced surgical time, reduced radiation exposure, and less evidence of neurological harm to the L5-S1 nerve root. The procedure was less complicated and easier for surgeons to learn. Its simplicity and speed were especially beneficial for obese patients.

## Introduction

Accounting for 3-8% of all orthopedic trauma, pelvic injuries are mostly the result of high-velocity injuries like traffic accidents or falls from a great height [1]. They are mostly associated with other systemic or bone fractures. Treatment of this type of injury has always been a subject of debate. Because of the destruction of the anterior and posterior pelvic ring structures, simple conservative management will not give satisfactory results [2-5].

Traditionally, doctors manage anterior pelvic ring fractures with plating techniques, which ensure anatomical reduction and stability. Because of the increased morbidity, mortality, blood loss, and prolonged surgical duration of these techniques, doctors now use a minimally invasive, anterior, subcutaneous, internal pelvic fixator (INFIX). This new technique involves subcutaneous placement of titanium rods and pedicle screws on the pelvis to ensure structural stability of the anterior ring [6].

Surgeons routinely prefer percutaneous sacroiliac joint screw fixation out of the many techniques that exist for posterior pelvic ring fractures. Despite the widespread use of this technique with INFIX, intraoperative complications and technical difficulties, including a high probability of error and difficulties in positioning the C-arm and achieving proper angles, pose challenges. In day-to-day clinical practice, we encountered problems with this procedure [7,8]. Positioning the screws in obese patients is technically demanding and difficult because other associated systems are involved, patients cannot be placed in a prone position, and unstable pelvic ring fractures might occur under abnormal stress when the anterior pelvic ring is not fixed. Moreover, a steep learning curve, loss of motion segments, persistent pain, and implant-related complications pose further challenges.

This study sought to evaluate the functional outcome of INFIX with and without sacroiliac joint screw fixation in unstable pelvic ring fractures. We used the Young and Burgess classification to classify fractures and a standard anteroposterior view of the pelvis with both hips. Special X-rays may be necessary to identify all the fracture segments. X-rays with inlet and outlet obturator and iliacus views may be necessary to plan the mode of operative management. CT scans are often required for exact bony reconstruction and MRI for soft tissue injuries [9].

## Materials and methods

We selected 20 patients, including 17 males and 3 females, with an average age of 42.9±7.1, and followed up with them for six months retrospectively. We identified patients by searching the hospital's database for people who had undergone INFIX surgery after approval from the Institutional Ethics Committee of Government Medical College Aurangabad (approval number: IEC/GMCA/226/2024). Retrospectively, we collected detailed information on fracture characteristics and surgical intervention from the case sheets of patients with unstable pelvic ring injuries who presented to a government tertiary care center in Maharashtra, India, between January 2023 and January 2024. We divided the 20 patients we selected into two groups of 10 each. One group underwent INFIX surgery with screw fixation (group A), and the other group received INFIX without screw fixation (group B). Subsequently, we recorded the following data from their case files: length of hospital stay, days spent in the ICU (if any), operative time, intraoperative blood loss, postoperative time, postoperative complications, and number of weeks for radiological signs of union.

Initially, the doctors stabilized the patients hemodynamically and treated injuries to other systems. Usually, patients need 5 to 10 days to settle. Doctors managed the fracture with pelvic binders, traction, bed rest, and bed sore prevention. Once the initial preoperative workup was complete, the doctors classified the fracture pattern according to the Young and Burgess classification system. They developed an operative plan after a thorough study of all X-rays and CT scans and an examination of the patient’s skin condition and general well-being. We followed up with the patients retrospectively for six months, using the Majeed score to grade functional outcomes. The Majeed score uses five criteria for functional assessment after pelvis injuries such as pain, sitting, standing, sexual intercourse, and work. These criteria are graded as excellent (78-80), good (70-77), fair (60-69), and poor (<60) [10].

Our inclusion criteria consisted of patients with unstable pelvic ring injuries, all closed pelvic ring injuries, all anterior pelvic ring fractures with sacroiliac joint disruption (unilateral or bilateral), an age of more than 18 years, and patients who gave written consent for surgery. The exclusion criteria consisted of patients with open pelvic fractures, fractures associated with acetabulum, patients with associated abdominal pathologies, patients with a bad skin condition, an age younger than 18 years, failure or refusal of the patient to give written consent for surgery, and a Glasgow Coma Scale (GCS) score of less than eight due to involvement of other systems. We analyzed the data to compare the functional outcomes between the two groups. Using a chi-square test with a p-value of less than 0.05, we determined the statistical significance of the differences we observed.

Operative procedures

INFIX, or internal fixation, is a treatment modality for anterior pelvic ring injuries that involves the insertion of polyaxial pedicular screws from the anterior, inferior iliac spine into the supra-acetabular region, directed toward the sacroiliac joint on both sides. Essentially, the surgeon uses the teardrop view as a guide to identify the anterior and inferior iliac spines [11].

**Figure 1:**
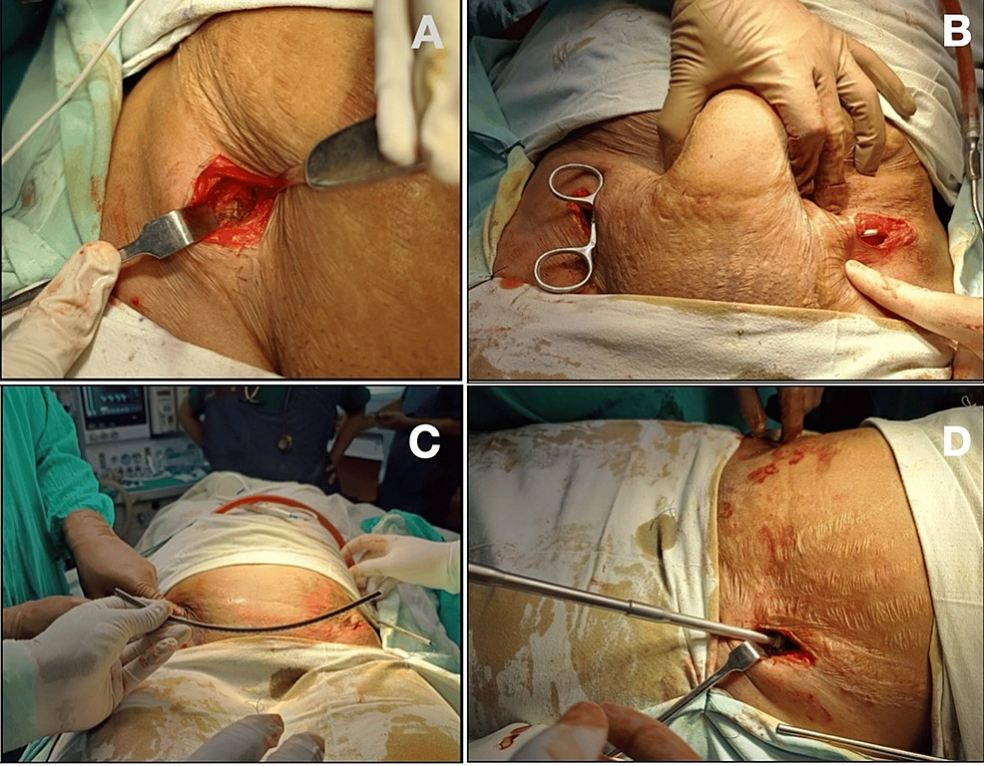
(A) A mini-incision taken over the anterior inferior iliac spine; (B) the creation of a subcutaneous tunnel; (C) pre-contouring of the rod; (D) subcutaneous placement of the rod and tightening of both ends

After making a small incision, the surgeon carries out a blunt dissection, creating a plane between the sartorius and tensor fascia lata to reach the anterior inferior iliac spine (Figure 1). Using an entry awl directed toward the supra-acetabular region, the surgeon gains entry with the help of c-arm guided shoots in an obturator inlet oblique view (Leeds view) and obturator and iliacus outlet view [12,13]. The surgeon then carefully inserts a polyaxial pedicular screw, usually 6.5-8.5 mm in diameter and 80-110 mm in length, and allows it to find its own path. After achieving entry, the surgeon keeps the pedicular screw around 20-25 mm above the cortex, maintaining a rod-to-bone distance [14].

The surgeon then carries out the same procedure on the opposite side and connects the two screws with a subcutaneous spinal rod, adequately contouring the rod to prevent intra-abdominal pressure symptoms. The surgeon also cuts the spinal rod cut short, ensuring there is no irritation to the lateral femoral cutaneous nerve. The patient receives pelvic compression and traction to achieve acceptable pelvic alignment, and the surgeon tightens the screws. The rod-to-symphysis distance was maintained at <40 mm to prevent lateral cutaneous nerve damage [14].

In sacroiliac joint screw fixation, the entry point is taken anterior to S1 and inferior to the iliac cortical density (ICD), which is parallel to the sacral alar slope, usually slightly caudal and posterior. The ICD thus marks the anterosuperior boundary of the safe zone for an iliosacral screw, which may injure the L5 nerve root if it penetrates this cortex. The surgeon taps a guide wire 2-3 mm or drills it into the planned entry point of the screw. The surgeon continuously checks the position under true anteroposterior and lateral views and confirms it with pelvic inlet and outlet views. Once it is confirmed, the surgeon further advances the guide wire to the contralateral lateral border of the first sacral body. In case of comminution, the surgeon uses fully threaded screws to avoid overcompression of a sacral fracture.

## Results

We selected 20 patients, including 17 males and 3 females, with a mean age of 42.9±7.1 years. The main mode of injury was road traffic accidents (17 cases), followed by falls from height (3 cases). We classified pelvic fractures according to the Young and Burgess classification system. Nine patients had anterior-posterior compression-II, five patients had anterior-posterior compression-III, and six patients had lateral compression-II. Ten patients received posterior fixation with a sacroiliac joint screw along with anterior INFIX. The other 10 patients received anterior INFIX alone. Table 1 shows the distribution of different types of fractures across both treatment groups. No significant difference exists between the types of fractures the surgeries treated.

**Table 1:** Distribution of the study subjects according to treatment and fracture classification INFIX: internal pelvic fixator; SI: sacroiliac

Fracture classification (Young and Burgess classification)	Number of patients-INFIX with SI joint screw	Number of patients-INFIX without SI joint screw	p-value
Anterior-posterior compression-II	5	4	0.85
Anterior-posterior compression-III	2	3	
Lateral compression-II	3	3	

Table 2 compares the clinical and biomechanical outcomes of INFIX with and without sacroiliac screw fixation for unstable pelvic fractures. No significant difference exists between the ages of the study participants in both treatment groups.

**Table 2:** Comparison of INFIX without SI joint screw and INFIX with SI joint screw *p-value < 0.05, statistically significant SD: standard deviation; INFIX: internal pelvic fixator; SI: sacroiliac

Variable	INFIX with SI joint screw (mean±SD)	INFIX without SI joint screw (mean±SD)	p-value
Age	41±6.83 years	44.7±7.3 years	0.25
Time till surgery	5.7±1.42 days	4.7±1.42 days	0.098
Procedure time	2.25±0.35 hours	1.05±0.23 hours	<0.01*
Intraoperative blood loss	113.5±70.75 ml	100±60.92 ml	0.51
Radiological union	4.6±0.7 months	4.5±0.7 months	0.73
Hospital stay	21.6±4.8 days	12±3.4 days	<0.001*
Functional assessment (Majeed score)	77.2±5.36	78±5.66	0.071

Two patients in the INFIX-alone group needed an ICU stay of an average of 3±6 days (a range from 6 to 12 days), while five patients who received INFIX with a screw required ICU admission. The reduction of fracture and bone union was satisfactory, and there was no internal fixation loosening or fracture reduction lost in both groups. No patients in either group showed signs of lateral femoral cutaneous nerve neuralgia. Two patients in the INFIX-with-screw group showed signs of superficial infection, which subsided with IV antibiotics. No patients in either group showed signs of deep infection.

The time until surgery was similar for both patients who received INFIX without sacroiliac screw fixation and patients who received INFIX with sacroiliac screw fixation (4.7 and 5.7 days, respectively). The time length of the procedure was significantly higher in INFIX with sacroiliac screw fixation (2.25 hours) than in INFIX without sacroiliac screw fixation (1.05 hours), with a p-value of <0.01. Although intraoperative blood loss was higher in patients who underwent INFIX with sacroiliac screw fixation (113 ml), it was not statistically significant compared to INFIX without sacroiliac screw fixation (100 ml).

The time required for the radiological union was also comparable in both treatment groups, at 4.5 months for INFIX without a sacroiliac screw and 4.6 months for INFIX with a sacroiliac screw. Patients who underwent INFIX without sacroiliac screw fixation had a significantly shorter hospital stay of 12 days compared to 21.6 days for patients who received INFIX with sacroiliac screw fixation, with a p-value of <0.001.

**Figure 2:**
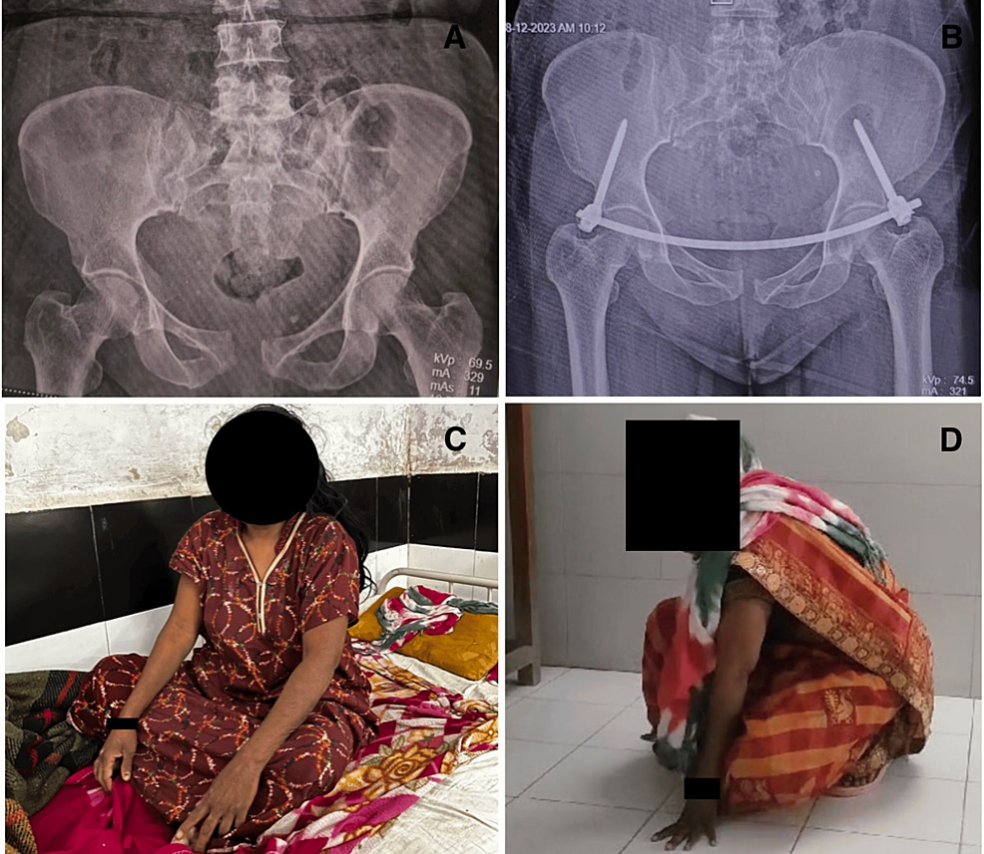
(A) Preoperative X-ray showing APC-II fracture; (B) postoperative X-ray INFIX; the patient sitting cross-legged (C) and squatting (D) six months after surgery APC: anteroposterior compression; INFIX: internal pelvic fixator

Functional assessment using the Majeed score was also comparable at the end of six months and did not show any significant variations. One patient from the INFIX-with-screw group experienced hypoesthesia in the L5/S1 dermatome and was treated with medical management, whereas no patients from the INFIX-only group reported such complaints. At the time of discharge, all patients could sit by the bedside with 90 degrees of flexion at the hip joint and a complete range of motion at the knee joint. The patients were mobilized on day 2, and thus, none of the patients in either group suffered from deep vein thrombosis or bed sores. Figure 2A shows a preoperative X-ray, Figure 2B shows a postoperative X-ray, and Figures 2C, 2D show the patient sitting cross-legged and squatting six months after surgery.

**Figure 3:**
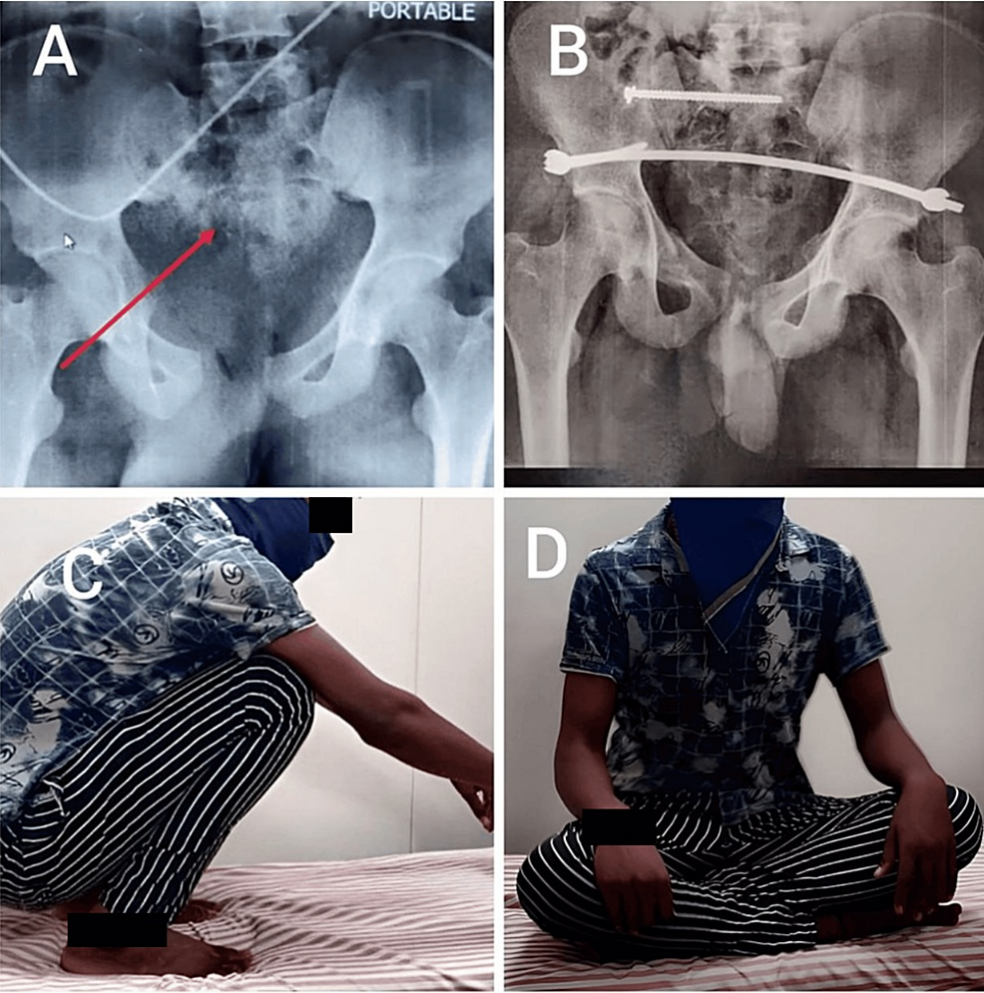
(A) Preoperative X-ray showing APC-II fracture; (B): postoperative X-ray of INFIX with SI joint screw; the patient squatting (C) and sitting cross-legged (D) six months after surgery INFIX: internal pelvic fixator; SI: sacroiliac

Figure 3 shows preoperative (A) and immediate postoperative (B) X-rays and the patient squatting (C) and sitting cross-legged (D) six months after surgery.

## Discussion

Pelvic ring fractures are mostly due to high-velocity trauma and are often associated with other systemic and bony injuries that require treatment. The ideal surgical techniques for anterior and posterior pelvic fractures have always been a subject of debate. Each technique has drawbacks and complications. Because of its modern advancement and cosmetic importance, we prefer anterior subcutaneous internal fixation (INFIX) for unstable, anterior pelvic ring fractures.

Many studies report INFIX has a satisfactory functional outcome comparable to that of anterior pubic plating. Previously, unstable pelvic fractures were treated with pelvic external fixators (EXFIX), but this treatment has now been confined to grade three compound, unstable pelvic ring fractures. INFIX results in reduced operative time, less blood loss, simplified postoperative care, and a lower risk of iatrogenic vascular injuries. INFIX is suitable for young female patients of childbearing age because it will not affect childbirth.

After more than a decade of clinical practice, INFIX has proven to be an efficient and effective method, offering good biomechanical stability and positive clinical outcomes. It is now considered an alternative treatment option for anterior pelvic ring fractures. Vaidya et al. were of the opinion that INFIX is a viable alternative in treating fractures of the pelvic ring in obese patients, in whom the rods of the EXFIX will compress the abdomen [15]. Posterior pelvic ring fractures are common with anterior ring fractures. Out of many available operative techniques, percutaneous sacroiliac joint screw fixation is the most common. Along with INFIX, it will stabilize both the anterior and posterior portions of the pelvis. The surgeon can also achieve a rigid and vertically stable fracture.

Most of the patients had associated abdominal or other bony involvement, so conventional prone positioning was not possible. In the supine position, especially in obese patients, inserting the guide pin is technically challenging. The surface of the operating table and other artifacts hinder proper visualization of the angle, thereby increasing the chance of malpositioning the screw. The long pedicular screws surgeons use (with a size of 70-110 mm) start from the anterior, inferior iliac spine, which is part of the anterior column of the pelvis. They fix these to the strongest part of the pelvis posteriorly, which gives reasonable stabilization to the posterior pelvic ring.

Because these are high-velocity injuries, skin conditions often are unfavorable for open or even percutaneous procedures, increasing the chance of deep infections. Patients also had complaints about persistent back pain after surgery. In a 2023 meta-analysis of percutaneous sacroiliac screw fixation, Alzobi et al. noted 6% had malpositioned screws, 3% had persistent pain, 2% had L5-S1 nerve injury, and 5% underwent revision surgery (screw removal for persistent pain) [16]. Intraoperative complications such as intra-pelvic entry of the guide needle and injury to the L5-S1 nerve were also common.

The disadvantages of this technique are that it creates a rigid and stiff pelvic complex, exposes the patient to more radiation, and exhibits a wide margin of error. Additionally, the technique is difficult to perform in obese patients, and operative time is prolonged, resulting in prolonged morbidity. Anatomical variations in the pelvis often make the technique technically demanding [17].

This study has a few limitations. The sample size is small, and the study is retrospective. We followed up with patients for only six months. A longer study with a follow-up period of at least two years would provide better information about the long-term effects. Also, large multicentric, prospective, randomized, and controlled studies will be required in the future.

## Conclusions

We studied 20 patients suffering from unstable pelvic ring fractures who underwent INFIX surgery with and without a sacroiliac joint screw and followed up for a period of six months retrospectively. Although this was a short-term study, we conclude INFIX with a sacroiliac joint screw showed a comparable functional outcome compared to INFIX without a sacroiliac joint screw.

The group receiving INFIX without a screw had better results in terms of reduced surgical time, reduced radiation exposure, and less evidence of neurological harm to the L5-S1 nerve root. The procedure was easier for surgeons to learn and less technically complicated, and its simplicity and speed were especially beneficial for obese patients.
